# Multilevel Social Mechanisms of Post-Disaster Depression

**DOI:** 10.3390/ijerph18020391

**Published:** 2021-01-06

**Authors:** Tim R. Wind, Ichiro Kawachi, Ivan H. Komproe

**Affiliations:** 1 Foundation Centrum ‘45, Oegstgeest, The Netherlands|Partner in Arq Psychotrauma Expert Group, Nienoord 5, 1112 XE Diemen, The Netherlands; 2 Harvard Medical School, 677 Huntington Avenue, Boston, MA 02115, USA; ikawachi@hsph.harvard.edu; 3 HealthNet TPO, Department of Research and Development, 1074 VJ Amsterdam, The Netherlands; ivan.komproe@hntpo.org; 4 Faculty of Social and Behavioral Sciences, Utrecht University, 3508 TC Utrecht, The Netherlands

**Keywords:** social capital, depression, social impact of flood, multilevel modeling, England

## Abstract

This exploratory study empirically shows how community social capital is related to post-disaster depression, whereas most disaster mental health research has focused on posttraumatic stress disorder. We tested the validity of earlier found multilevel social and individual mechanisms of posttraumatic stress for symptoms of post-disaster depression. We used data (n = 231) from a community study after a flood in Morpeth (2008), a rural town in northern England. At the salutary community level, our multilevel analyses showed that, in communities with high social capital, individuals employ less individual social support and coping effort, which protects individuals from developing symptoms of depression. Yet, on the ‘dark’ individual level of our model, we found that perceiving the disaster as less traumatic after a year was related to more feelings of depression in contrast to previous findings for posttraumatic stress. Our explanation of this finding is that, when the appraisal of the disaster as threatening fades into the background, individuals may perceive the full scope of the disaster aftermath and start to feel depressed. We also found that more social support is related to more depression. Although depressed people may attract or receive more social support, this social support can paradoxically become disabling by reinforcing a sense of dependence, thereby undermining self-esteem and leading to feelings of helplessness. Our results imply that to curb post-disaster depression, boosting community level social capital may be an important starting point for building resilience. At the same time, interventionists need to identify risk groups for whom the stressful experience becomes less intrusive and who experience the burden of dependency on an unequal relationship with ones’ social inner circle.

## 1. Introduction

In the past decade, the World Health Organization (WHO) has taken a leading role in the development, study, and dissemination of best-practice materials and provision of technical field support for Mental Health and Psychosocial Support (MHPSS) operations. Based on the experience from these activities, a clear need emerged for guidance on developing MHPSS programming from a Disaster Risk Reduction (DRR) perspective. Strong arguments have been made for linking MHPSS and DRR activities and for shifting paradigms in the field of MHPSS towards preparedness and prevention, yet without much evidence based response [1]. The current study focuses on social capital as an explicit link between DRR and MHPSS.

Literature on the salutary aspects of community social capital has grown, moving from early attempts to define the concept of social capital and its associations with (mental) health and well-being [2] to empirical research on how community social capital is related to (mental) health and well-being [3,4,5]. There are several definitions of social capital, but in general social capital is defined as ‘the resources an individual can draw on through his or her social networks and the value ascribed to these resources by the individual’ [2,6,7].

Most disaster-related mental health research in the last thirty years has focused on posttraumatic stress disorder [4,5,8,9,10]. An earlier empirical study found that higher social capital was associated with more adequate and efficient employment of individual coping strategies—including the mobilization of the social support of friends and family [4,10]. This parsimonious deployment of individual psychosocial resources decreases the association between the traumatic appraisal of the disaster and posttraumatic stress [10]. Hence, these findings suggested that, in communities with high social capital, affected individuals are more resilient to symptoms of posttraumatic stress [11].

There is, however, scanty empirical evidence showing how community social capital is related to post-disaster depression. Research suggests that the disaster context may play a more prominent role for symptoms of depression [12,13,14]; posttraumatic stress is by definition related to the individual experience of one or more traumatic events, whereas depression is by nature reciprocally related to the social context. On the one hand, a lack of social ties can be a consequence of depressive symptoms, as depressed individuals become more inactive and consequently participate less in their social circles. On the other hand, after most disasters, social contexts lose their salutary ‘buffer function’ against depressive symptoms as traditional social support systems do not function as before because family members or other members of the social network may be dispersed, or may be in need of support themselves, or may even have died, and social routines are disrupted due to home loss [15,16]. Consequent social isolation and loss of social ties are among the most potent predictors of depressive symptoms [17].

In this paper, we test the robustness of earlier found multilevel social and individual mechanisms of posttraumatic stress for symptoms of post-disaster depression [10]. The study was conducted one year after a flood in Morpeth, a small town located in northern England. In September 2008 the residents of Morpeth were confronted with its worst flood in half a century, which left great material damage. The understanding of how (damaged and disrupted) social mechanisms are associated to post-disaster depression will inform policy makers and interventionists to curb symptoms of depression in the aftermath of disasters [10].

## 2. Method

We obtained a list of flooded premises that comprised 757 households from the local authorities in Morpeth. In August and September 2009, we approached these households. In cases where respondents were absent, the addresses were revisited twice. Ninety respondents refused to participate in the survey due to a lack of time. Despite the migration of some residents because their houses were still not livable (41 respondents), the absence of households members at the time of study (390 respondents), and too many missing values among five of the respondents, we were able to administer the interview to 231 respondents (72% of the approached respondents and 30.6% of the total address list participated in the study). The demographics of the samples are depicted in Table 1.

A local research firm conducted the survey with experienced local surveyors under supervision of the local principal investigator. The surveyors received one-day training in the administration of the questionnaire. Written informed consent was obtained from the participants after an introduction and explanation of the study purpose by the surveyors. The ethical approval of the study was obtained from Northumbria University.

### 2.1. Measurement of Variables

Depression. Symptoms of depression were assessed by the subscale depression of the Hopkins Symptom Checklist-25 [HSCL-25] [18]. The period of reference is the last month. The depressive symptoms score is the average of the 15 depression items of the subscale. The respondent is asked to report how much he or she has been bothered by each item during the last month on a 5-point scale ranging from 1 = “not at all” to 5 = “extremely”. The internal consistency (Cronbach’s alphas) of the Depression scale in this study was 0.69.

#### 2.1.1. Community Variables

Social Capital. We selected the SA-SCAT (Shortened and adapted Social Capital Assessment Tool) [19] to measure social capital. Some items of the SA-SCAT were adapted to improve the relevance for the local context [10]. Structural capital was measured by eight items with a four point response format. Cronbach’s alpha was 0.74. Cognitive social capital was measured by seven statements with a four point response format. Cronbach’s alpha was 0.76.

Collective Efficacy. Collective Efficacy comprises five items with a five-point response format [20]. The Collective Efficacy scale measures the willingness to intervene (collectively) in neighborhood-threatening situations. Residents were asked about the likelihood that neighbors could count on assistance in five specific community situations. Cronbach’s alpha was 0.92.

Several scholars [2,20,21] have advocated the inclusion of objective indicators of social capital because social capital measured by self-report questionnaires is partly determined by the perception of individuals. Sampson et al. [20] showed that Residential Stability can be used as an indicator for social interactions in a neighborhood. Residential Stability in this study was measured by the response options: rented (1), owned with mortgage (2), owned outright (3).

#### 2.1.2. Individual Level Variables

Disaster Property Loss was defined as an indicator of the severity of the individual disaster experience. The variable was measured by four questions with a five point response format: To what extent did you experience damage or loss to: (1) the structure of your house, (2) the contents and belongings of your house, (3) personal belongings with sentimental value, (4) your car. The total Property Loss score was used in the analyses.

Primary Appraisal. Primary appraisal, the perceived threat of the situation, was measured by the question “How traumatic was the flood for you at the time?” Respondents could indicate their answers on a five-point response format ranging from “not at all” [1] to “extremely” [5].

Coping Effort. Coping Effort is defined as the extent to which a variety of coping strategies are employed to deal with an experienced stressor. We used a questionnaire with six items with a five point response format that assessed individual coping [22]. The items referred to the strategies Avoidance, Reappraisal, Religion, Active cognitive coping, Active behavioral coping, and Seeking social support. Cronbach’s alpha was 0.86.

Social Support. The Social Support Scale of Harper and Kelly [23] was used to assess Social Support. Respondents were asked to indicate how often they received ten types of social support on a five point response format. Cronbach’s alpha was 0.72.

### 2.2. Statistical Analyses

It has been argued [24] that ecological associations are best explored using data from small areas such as the ‘home patch’ that constitute a homogeneous community. Thomas [24] claimed that the postcode unit is a rough proximate of the geographical area where the key social interactions take place in England. In this study, the individual scores on community variables were aggregated to the postcode units.

In a previous paper [10], we modeled the within and between-level variance of both the individual and community variables, simultaneously through a stepwise procedure. The community-level random effect of the intercept was assumed to be normally distributed with a mean of zero.

Empty model: We examined the community-level variance in posttraumatic stress without including any explanatory variables.

Model 1A–1B: Model 1A is the model with the best fit. In model 1B, we examined the direct cross-level association between the community variables and posttraumatic stress without including the individual variables.

Model 2: In model 2, we examined the direct pathways from the community variables to posttraumatic stress in addition to the explained variance of the individual Best Fitting (BF) model.

Model 3A–3C: In model 3A to 3C, we examined the direct cross-level pathways between each separate community variable and the individual variables, and the cross-level interaction terms of the community variables and the relationships in the individual BF model.

Model 4: In model 4, we tested the total multilevel model that included all significant structural equation relationships at the individual and community level in the previous models (1–3) and additional hypothesized pathways from Structural Social Capital to Cognitive Social Capital and Collective Efficacy, and Cognitive Social Capital to Collective Efficacy. Additionally, we tested the hypothesis that Residential Stability and Income facilitated access to social capital.

In this study, we tested the robustness of the final multilevel structural equation modeling for post-disaster depression (ML-SEM; model 4). The multilevel modeling was based on (i) the likelihood of the estimates (significance < 0.05), (ii) the degree of support for the estimates in the literature (i.e., theoretical value), and (iii) a set of model fit indices. We evaluated the fit of the ML-SEM models by two fit indices: (1) Likelihood Ratio Test (LRT) for nested-model fit, and (2) Akaike Information Criterion (AIC). Lower values of the LRT and AIC indicate a better fit [24,25]. Multilevel structural equation modeling was executed using MPLUS 8 software.

## 3. Results

### 3.1. Demographics

Table 1 depicts the individual demographic information of the sample. The study sample contains 231 individuals nested within 59 postcode units with an average cluster size of 3.91 individuals per postcode unit.

### 3.2. Multilevel Structural Equation Modeling

The intra-cluster correlation for the variable Depression across postcodes was 0.21.

**Figure 1:**
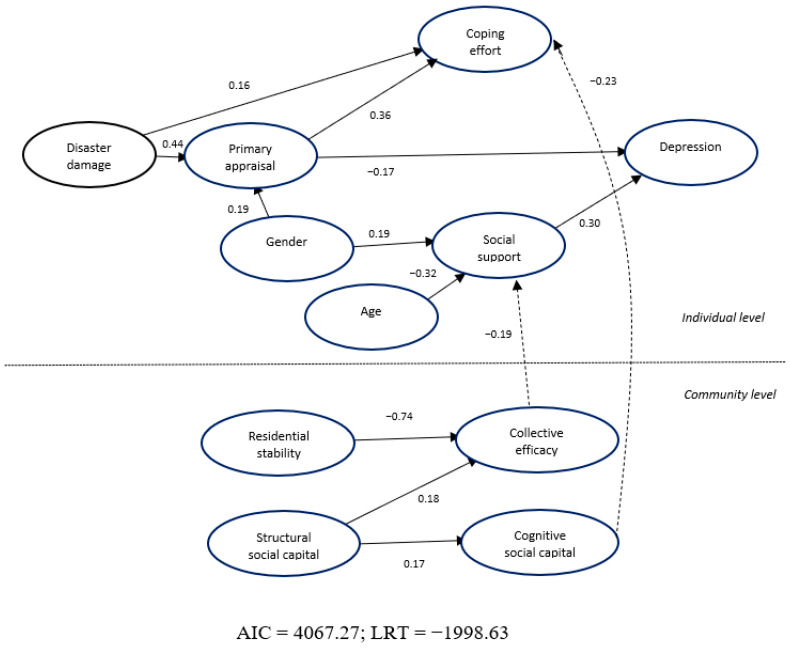
The multilevel structural equation model.

The model is depicted in Figure 1. The structure of the individual model that was tested is similar to the model that associates the study variables with posttraumatic stress [9]. In this study, the variable Depression was related to Social Support (*β =* 0.30; *p* < 0.001) and Primary Appraisal (*β = −*0.17; *p* < 0.01) but not to Coping Effort (*p* > 0.05). Social Support was related to Gender (β = 0.19; *p* < 0.01) and Age (*β = −*0.32; *p* < 0.001). Coping Effort was related to Primary Appraisal (*β =* 0.36; *p* < 0.001) and Disaster Damage (*β = 0*.16; *p* < 0.01). Primary Appraisal was related to Gender (*β =* 0.19; *p* < 0.01), implying that women appraised the disaster as more traumatic, and Primary Appraisal was related to Disaster Damage (*β =* 0.44; *p* < 0.001).

At the community level, Residential Stability (*β* = −0.74; *p* < 0.001) and Structural Social Capital (*β* = 0.18; *p* < 0.001) were related to Collective Efficacy. Structural Social Capital was related to Cognitive Social Capital (*β =* 0.17; *p* < 0.001).

There were two significant cross-level relationships: Collective Efficacy was associated to Social Support (*β =* −0.19; *p* < 0.05) and Cognitive Social Capital was related to Coping Intensity (*β =* −0.23; *p* < 0.01).

## 4. Discussion

Recently, Gray et al. [1] highlighted a clear need for guidance on developing MHPSS programming from a DRR perspective. Yet, DRR activities have not been narrowly defined and there has been a lack of evidence linking DRR activities with MHPSS. This study attempted to bridge that gap by revealing the link between social capital (an unstructured DRR component) and post-disaster depression as an important MHPSS issue and intervention target.

Equal to our previous study on posttraumatic stress [10], on the salutary community level, our multilevel model revealed that community social capital is related to post-disaster depression problems (e.g., symptoms of depression) via individual social support. Specifically, our findings showed that, in a neighborhood with more collective efficacy, people mobilize less social support and, hence, people may become less dependent on social support from their social network (e.g., friends and family). In a community with high social capital, such “conservation of individual social support” was associated with less suffering from symptoms of depression [17]. In other words, the social context was health sustaining by its association with individual social support. In our study, older people received less social support and seemed to be more isolated. The beneficial effect of the collective efficacy to address disaster-related demands on the individual level was larger in communities with less residential stability. That is, in neighborhoods where more houses are rented, individuals benefit more from the collective to address the consequences of the disaster. Although high levels of cognitive social capital were associated with lower coping efforts, these coping efforts were not related to depressive symptoms in our multilevel model.

In contrast to our previous study on posttraumatic stress [10], on the ‘dark’ individual level of our model, we found that perceiving the disaster as less traumatic after a year was related to more feelings of depression. That is, when the traumatic appraisal of the disaster fades into the background, individuals may perceive the full scope of the disaster aftermath (e.g., material damage or loss of livelihood) and start feeling depressed. Media attention and generous outsiders often abandon communities when victims discover that the struggle to rebuild their physical and social environment has just begun [12]. Similarly, Norris et al. [13] showed that, whereas intrusions and arousal abated after the disaster, symptoms of depression increased for a substantial minority at the same time. Contrary to our previous multilevel model for posttraumatic stress [10], this time we found that more social support is related to more depressed symptoms; whereas depressed people may attract or perceive more social support, this social support can paradoxically become disabling by reinforcing a sense of dependence, thereby undermining self-esteem and leading to feelings of helplessness [16].

All in all, our findings imply a nuanced notion beyond the black-and-white idea that social capital interventions (e.g., community interventions that aim to rebuild the damaged social fabrics on the individual and community level simultaneously) naturally exert the strongest effects on mental health outcomes [26,27]. Rather, to curb post-disaster depression, community level social capital interventions may be an important salutary starting point, as social capital facilitates and might bolster the effect of ‘perceived’ social support. Importantly, community social capital interventions have additional cost efficient benefits that impact the health of individuals targeted by the interventions as well as people who are connected to these individuals (i.e., spillover effects, also known as “collateral benefits”). Yet, our results imply that, at the same time, interventionists need to identify the individuals for whom the traumatic experience becomes less intrusive and the full consequences of the disaster start to sink in, accompanied by feelings of depression. Such individuals who acquire much social support from friends and family may consequently feel the burden of dependency on an unequal relationship with ones’ social inner circle. These individuals need to be targeted for additional individual psychosocial interventions.

## 5. Limitations

This study has some potential limitations. First, the cross-sectional design of our study and the inherent absence of data on pre-flood mental health did not allow for causal inferences of disaster related distress to the flood specifically. We tried to overcome these limitations by focusing the mental health questions on the particular experience of the flood. Furthermore, the technique of Ml-SEM enabled us to specify (uni-directional) pathways within the model and provided us insight into the social mechanisms related to disaster-related distress. Second, the response rate was relatively low, and it is not clear to what extent the study sample is representative of the population of affected households in Morpeth. Third, the relatively high age and the particular composition of the study sample may hamper the extrapolation of our study results to other disaster-affected populations. We attempted to partly overcome this limitation by modeling the age variance in our study. Fourth, the aggregation of individual scores to measure community variables (social capital and collective efficacy) allows for weeker conclusions than would the use of independent measures of community variables (such as the distance to social facilities). We recommend the use of independent measures of community variables in future studies.

## 6. Conclusions

This study demonstrated the role of community social capital for symptoms of post-disaster depression. We showed a salutary association of community social capital; our multilevel model showed that, in communities with high social capital, a disaster may be less demanding for individual psychosocial resources, and inherently individuals may suffer less from symptoms of depression. On an individual level, we showed that people with less traumatic experience and individuals who receive more social support from family and friends may comprise groups who are at risk of suffering feelings of depression after a year. Our findings have nuanced implications for post-disaster community interventions.

This study is an invitation for scholars to similarly dissect the social mechanisms of post-disaster mental health as this type of research remains rather scarce. We highlight that our study is cross-sectional by nature and therefore does not allow for causal inferences. To address this methodological issue, we urge scholars to undertake longitudinal studies on social mechanisms of disaster mental health.

## Figures and Tables

**Table 1:** Demographic characteristics of the study sample.

	Frequency (Percentage)
Gender	
Male	90 (39.0)
Female	141 (61.0)
Age group	
<18	0 (0)
18–24	4 (1.7)
25–39	20 (8.7)
40–64	69 (29.9)
>65	138 (59.7)
Religion	
Religious	197 (85.3)
None	34 (14.7)
Marital status	
Married	83 (35.9)
Single	40 (17.3)
Separated	6 (2.6)
Divorced	23 (10.0)
Widowed	73 (31.6)
Common law	6 (2.6)
Education *	
<High school	93 (40.3)
High School	58 (25.1)
Some college	13 (5.6)
College or post-graduate	51 (22.1)
Work	
Employed	75 (32.4)
Seeking work	12 (5.2)
Carer or looking after children/house	9 (3.9)
Student or on training scheme	2 (0.9)
Retired	133 (57.6)

* There were 16 missing values in the variable Education.

## References

[1] Gray B.L., Hanna F., Reifels L. (2020). The Integration of Mental Health and Psychosocial Support and Disaster Risk Reduction: A Mapping and Review. Int. J. Environ. Res. Public Health.

[2] Kawachi I., Subramanian S.V. (2006). Measuring and modeling the social and geographic context of trauma: A multilevel modeling approach. J. Trauma. Stress.

[3] Villalonga-Olives E., Wind T., Kawachi I. (2018). Social capital interventions in public health: A systematic review. Soc. Sci. Med..

[4] Wind T.R., Fordham M., Komproe I.H. (2011). Social capital and post-disaster mental health. Glob. Health Action.

[5] Wind T.R., Joshi P.C., Kleber R.J., Komproe I.H. (2014). The effect of the postdisaster context on the assessment of indi-vidual mental health scores. Am. J. Orthopsychiatr..

[6] Bourdieu P., Richardson J. (1986). The Forms of Capital. Handbook of Theory and Research in the Sociology of Education.

[7] Hurtado D.A., Kawachi I., Sudarsky J. (2011). Social capital and self-rated health in Colombia: The good, the bad and the ugly. Soc. Sci. Med..

[8] Norris F.H., Friedman M.J., Watson P.J., Byrne C.M., Diaz E., Kaniasty K. (2002). 60,000 Disaster Victims Speak: Part I. An Empirical Review of the Empirical Literature, 1981–2001. Psychiatry.

[9] Norris F.H., Friedman M.J., Watson P.J. (2002). 60,000 Disaster Victims Speak: Part II. Summary and Implications of the Disaster Mental Health Research. Psychiatry.

[10] Wind T.R., Komproe I.H. (2012). The mechanisms that associate community social capital with post-disaster mental health: A multilevel model. Soc. Sci. Med..

[11] Hikichi H., Aida J., Tsuboya T., Kondo K., Kawachi I. (2016). Can Community Social Cohesion Prevent Posttraumatic Stress Disorder in the Aftermath of a Disaster? A Natural Experiment from the 2011 Tohoku Earthquake and Tsunami. Am. J. Epidemiol..

[12] Nandi A., Tracy M., Beard J.R., Vlahov D., Galea S. (2009). Patterns and Predictors of Trajectories of Depression after an Urban Disaster. Ann. Epidemiol..

[13] Norris F.H., Tracy M., Galea S. (2009). Looking for resilience: Understanding the longitudinal trajectories of responses to stress. Soc. Sci. Med..

[14] Tsuboya T., Aida J., Hikichi H., Subramanian S., Kondo K., Osaka K., Kawachi I. (2016). Predictors of depressive symptoms following the Great East Japan earthquake: A prospective study. Soc. Sci. Med..

[15] Crighton E.J., Elliot S.J., van der Meer J., Small I., Upshur R. (2003). Impacts of an environmental disaster on psycho-logical health and well-being in Karakalpakstan. Soc. Sci. Med..

[16] Weems C.F., Watts S.E., Marsee M.A., Taylor L.K., Costa N.M., Cannon M.F., Carrion V.G., Pina A.A. (2007). The psychosocial impact of Hurricane Katrina: Contextual differences in psychological symptoms, social support, and discrimination. Behav. Res. Ther..

[17] Kawachi I., Berkman L.F. (2001). Social ties and mental health. J. Urban Health.

[18] Derogatis L.R., Lipman R.S., Rickels K., Uhlenhuth E.H., Covi L. (1974). The Hopkins Symptom Checklist (HSCL): A self-report symptom inventory. Syst. Res. Behav. Sci..

[19] Harpham T., Grant E., Thomas E. (2002). Measuring social capital within health surveys: Key issues. Health Policy Plan..

[20] Sampson R.J., Raudenbush S.W., Earls F. (1997). Neighborhoods and violent crime: A multilevel study of collective effi-cacy. Science.

[21] Almedom A.M. (2005). Social capital and mental health: An interdisciplinary review of primary evidence. Soc. Sci. Med..

[22] Mattlin J.A., Wethington E., Kessler R.C. (1990). Situational Determinants of Coping and Coping Effectiveness. J. Health Soc. Behav..

[23] Harper R., Kelly M. (2003). Measuring Social Capital in the United Kingdom.

[24] Thomas H., Weaver N., Patterson J., Jones P., Bell T., Playle R., Dunstan F., Palmer S., Lewis G., Araya R. (2007). Mental health and quality of residential environment. Br. J. Psychiatry.

[25] Mehta P.D., Neale M.C. (2005). People are variables too: Multilevel structural equations modeling. Psychol. Methods.

[26] Schölmerich V.L.N., Kawachi I. (2016). Translating the social-ecological perspective into multilevel interventions for family planning: How far are we?. Health Educ. Behav..

[27] Schölmerich V.L.N., Kawachi I. (2016). Translating the socio-ecological perspective into multilevel interventions: Gaps between theory and practice. Health Educ. Behav..

